# Reducing stigma and increasing workplace productivity due to mental health difficulties in a large government organization in the UK: a protocol for a randomised control treatment trial (RCT) of a low intensity psychological intervention and stigma reduction programme for common mental disorder (Prevail)

**DOI:** 10.1186/s12889-020-09054-0

**Published:** 2020-06-09

**Authors:** Nicola S. Gray, Helen Davies, Robert J. Snowden

**Affiliations:** 1 Department of Psychology, College of Human and Health Sciences, Swansea University, Swansea, Wales SA2 8PP UK; 2 Caswell Clinic, Swansea Bay University Health Board, Bridgend, UK; 3 Driver and Vehicle Licensing Agency (DVLA), Swansea, UK; 4 School of Psychology, Cardiff University, Cardiff, UK

**Keywords:** Randomised control trial, Prevail, Work-based intervention, Self-stigma, Stigma, Avoidant coping, Absenteeism, Presenteeism

## Abstract

**Background:**

Common mental disorders are the leading cause of workplace absences. While the reasons for this are multifarious, there is little doubt that stigma related to common mental disorder plays a large role in sickness absence and in poor help-seeking. Frequently both managers and staff are unsure of how to approach and intervene with mental health related problems. We have therefore devised a mental health intervention programme (Prevail) that aims to reduce stigma and to educate staff about evidence-based low intensity psychological interventions. These can be used by the individual, as well as in collaboration with managers via co-production of problem-focussed solutions, with the aim of improving mental health, reducing sickness absence, and increasing workplace productivity.

**Methods:**

This two-armed cluster randomised control trial (RCT) will evaluate the effectiveness of Prevail. Eighty managers at a large UK government institution (the DVLA) and their teams (approximately 960 employees) will be randomised into the active intervention group or control (employment as usual) arms of the study. All participants will be invited to complete a series of questionnaires related to mental health stigma, their current and past mental health, and their recent workplace productivity (absenteeism and presenteeism). All employees in the active arm will receive the Prevail Staff intervention, which covers stigma reduction and includes psychoeducation about evidence-based low intensity psychological interventions for common mental disorder. The managers in the active arm will also receive the Prevail Managers programme which covers communication skills, problem formulation, and problem-solving skills. The questionnaire battery will then be given to both groups again 4 weeks post training, and 12 months post-training. Official records of absenteeism from Human Resources will also be gathered from both active and control groups at 12 months post-training.

**Discussion:**

The treatment trial aims to evaluate if Prevail reduces mental health related stigma (of a number of forms), increases help-seeking behaviours, and increases workplace productivity (via decreased absenteeism and presenteeism).

**Trial registration:**

ISRCTN12040087. Retrospectively registered 04/05/2020.

## Background

Common mental disorder (CMD, i.e. anxiety and depression) are indeed common, contributing around 16–17% of the burden of adult disease in the UK [1]. They are also major factors in sickness absence from work [2–5]. This has significant negative outcomes for both the employer and for the economy due to lost productivity.

The effects of CMD in the workplace may be more prevalent than most statistics suggest. First, individuals that are later diagnosed with a CMD are found to have increased visits to General Practitioners for other health reasons compared to controls [6] and have an increased number of days sick leave prior to a diagnosis of mental health problems [7]. Thus, sick days taken due to mental health problems are often recorded as due to other (physical) problems. Further, women with a psychiatric diagnosis also have a greater incidence of sick-leave due to non-psychiatric reasons, such as gastro-intestinal diseases [5, 8]. While this may reflect some co-morbidity (or a side effect of treatment) it also might suggest that the reasons given for sick leave may not always be accurate and that non-mental health reasons may be given as the reason for illness when the reason is due to poor mental health.

There have been several studies of treatment and therapies designed to enable people with CMD to return to work. Standard CMD treatments, such as cognitive behavioural therapies and psychotropic medication have significant effects on symptom reduction, but do not have an impact on return to work and only modest effects on amount of sick leave [9]. Perhaps more effective results might be obtained if there were more workplace-based interventions that involved co-operative sickness management plans that involved both the person and their employer working together for the benefit of both.

Workplace interventions specifically target the problem as it affects the person’s ability to function in the workplace and involve the active involvement of the employee. However, such a process is likely to be challenging as the employee and employer may have different perspectives and aims [10]. Nevertheless, there is some, although mixed, evidence that work-based interventions can reduce sick leave due to CMD [11, 12].

### Prevail

Prevail is a multi-faceted programme aimed at reducing sickness absence and presenteeism due to CMDs. It involves two psychological interventions, both provided via group based intervention programmes. The first (Prevail-Staff) is for all employees within the organization and its aims are to improve knowledge about mental health, including knowledge of best-practice in low intensity psychological inventions and the theoretical premises underpinning such interventions. It also aims to reduce stigma related to mental health issues, and in particular self-stigma [13], and thus promote help-seeking behaviours both within and outside of the workplace. It covers: i) the basics of mental health literacy; ii) the normalization of CMD; iii) attempts to reduce stigma associated with CMD, with an emphasis on self-stigma; and iv) a plan of managing CMD within the workplace to reduce distress and work-place functional impairment. This includes situations when simple adjustments in work-based practice may greatly assist, when low intensity psychological interventions are appropriate, and when professional psychiatric or medical help may be required.

The second intervention (Prevail-Manager) is aimed at the managerial level within the workplace and is designed to teach managers a formulation-based approach to evaluation and intervention. Formulation refers to a process of providing an explanation for the presenting problem and differs from a “diagnosis” which is more categorical and refers to the actual CMD rather than the causes, or trigger-factors, of the CMD. The focus here is on understanding, active problem-solving, and co-production (where both the employer and the employee share the responsibility to plan and deliver the intervention within the work-place and both make a contribution and commitment to this plan [14]), with the aim of preventing sick leave and enhancing productivity.

### Aims and hypotheses

This Protocol presents the design of a cluster randomised control trial to examine the effects of Prevail. We will include three levels of outcome: (i) mental health literacy, with emphasis on levels of mental health stigma and self-stigma; (ii) behavioural data (sick leave and presenteeism); and (iii) measures of mental health and quality of life.

We hypothesise that the employees who have undergone the Prevail intervention programme (which encompasses both the Prevail-Staff intervention and the Prevail-Manager intervention programme), compared to the employment-as-usual (EAU) group of employees, will have: (i) reduced levels of mental health stigma and increased willingness to seek help for CMD; (ii) fewer sick days in the 12 months following the Prevail intervention programmes (Prevail-Staff and Prevail-Manager); and (iii) lower levels of presenteeism (as estimated via self-report). Mental health and quality of life measures will also be taken in order to be able to take a baseline measure of health economics for later translation to a cost-benefit analysis and health economics evaluation of the Prevail intervention relative to productivity savings.

## Methods/design

**Fig. 1:**
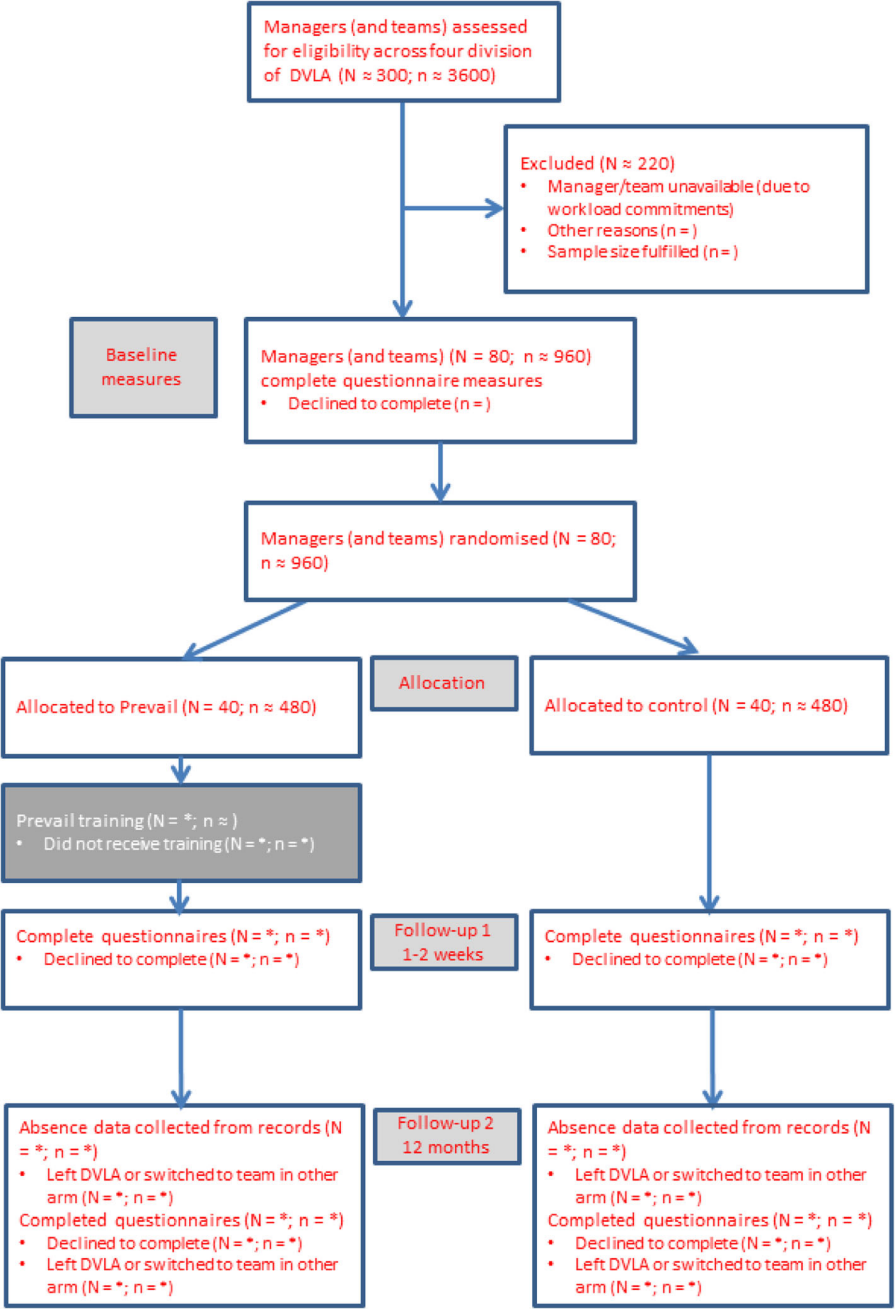
Consort Representation of Study

A CONSORT statement was used to describe the study [15]. The study design is a two-armed cluster randomised control trial of Prevail to decrease mental health stigma (with a specific focus upon self-stigma) and increase help-seeking behaviour. This, in turn, should decrease absenteeism and presenteeism (see Fig. 1). The setting is the Driver and Vehicle Licencing Agency (DVLA). The DVLA is the executive agency part of the Department for Transport. DVLA maintain the registration and licensing of drivers and vehicles in Great Britain. It employs around 6000 people mainly at its headquarters in Swansea, Wales, UK.

A clustered randomised control trial (RCT) design was deemed necessary as the Prevail programme addresses the two-way communication of mental health information and planning of active problem-solving and mental health interventions (and/or help seeking) between a manager and the employees within their team. Hence, both the manager and all members of their team had to be in the same arm of the intervention and the only way to achieve this is via a clustered RCT design. Hence, randomisation will take place at the level of the managers and will involve clusters of approximately 12 people (although this varies from division to division within the DVLA). However, the main outcomes (e.g. attitudes to mental health, absences from work) will be at the level of the individual employees of the DVLA and not at this cluster level.

### Recruitment and consent

Eighty managers across four divisions of the DVLA will be chosen by the DVLA to take part in the study on the basis of workload considerations. All managers will be given information about the aims and objectives of the study and will be asked by the DVLA to take part. However, participants will be blind as to whether they will be in the Prevail or Employment as Usual (EAU) group. These 80 managers will be managing approximately 960 employees.

Prior to the commencement of the Prevail intervention programme, managers and employees will be invited to a pre-intervention information session to explain the aims of the Prevail programme and be told that they have been selected as possible candidates for this psychological intervention. At this pre-session all participants (employees and managers) will be invited to complete the baseline evaluation measures. Completion of the baseline measures is voluntary and written informed consent will be taken at this time for those that wish to participate in the study and complete the evaluation measures. All participants and researchers are blind at this stage to as whether they are to be allocated to the Prevail (Active) group of Employment as Usual (Control) group. Any employees and managers who do not wish to participate in the research project will be free to leave the pre-intervention session without the need to justify their decision.

The 80 managers will then be allocated to the Active or Control arms of the study. Allocation will be random, but stratified by division and by the gender of the manager to ensure equal numbers within Active and Control groups across these variables. For managers and their teams in the Active arm of the study, the DVLA has mandated the Prevail intervention programme as part of their work commitments, but the evaluation of outcomes of this via the cluster RCT was not mandated.

For the two follow-up data collection waves: post-Prevail (termed wave 2) and 12-month follow-up (termed wave 3), the DVLA will mandate that staff in both the Active and Control arms of the research design attend follow-up sessions. At these sessions, participants will be briefed as to the progress of the Prevail intervention trial and will be invited to complete the psychometric measures as evaluation of the intervention programme. Once again, completion of the measures is voluntary and written informed consent will be taken at this time for those that wish to participate in the evaluation. Those participants who do not so wish are free to leave prior to completion of the psychometric measures.

Data from Human Resources (HR) records for the staff and managers in the study will be processed by the DVLA Human Resources staff. This data will only be communicated to the researchers at a group level (e.g., average number of sick days in the Prevail group as compared to the Control group over the last 12 months), separated by gender and age, etc., in order to ensure that the sickness absence data is anonymous to the research team. If the group size for any specific data (e.g., average number of sick days in the Prevail group over the last 12 months for females aged 60+, etc.) falls below an N of 10 this data will not be communicated to the research team in order to protect the identities of the staff group.

### Randomisation

The randomisation will take place at the level of managers (*N* = 80). Managers will be stratified by division of the DVLA [Information Technology Services (ITS), the Contract Centre (CC), Casework and Enforcement Group (CAEG), and Input Services Group (ISG)] and by the gender of the manager. This is to ensure that these variables are equally distributed across the two arms of the study. A computer-generated random sampling procedure will be used to ensure unbiased allocation to each group: https://www.sealedenvelope.com/.

### Eligibility criteria

#### Inclusion criteria

Eligible participants can be included if they meet the following criteria:

- Employed at the DVLA and aged 18 to 70.
- Understand written and spoken English or Welsh.

#### Exclusion criteria

- None.

### Intervention

All employees in the intervention group will engage in the Prevail-Staff psychological intervention programme in addition to the employment as usual facilities provided by the DVLA (see below). This involves attendance at a one-day intervention programme that incorporates a number of psychological techniques designed to: i) improve knowledge about mental health and cover the basics of mental health literacy; ii) enhance the normalization of common mental disorder; iii) reduce stigma associated with common mental disorder, with an emphasis on self-stigma; and iv) assist staff to learn how to formulate a plan of managing common mental disorder within the workplace to reduce distress and workplace functional impairment. The Prevail programme includes detailed information about evidence-based low intensity psychological interventions for common mental health disorders. This includes intervention strategies for depression, anxiety, stress, and distress (including bereavement). The Prevail programme also actively encourages disclosure of mental health difficulties and appropriate help seeking behaviour. A Train the Trainer approach [16] will be taken in which six employees will be chosen as trainers and educated on the provision of Prevail-Staff by members of our research team (Gray and Snowden). The Prevail trainers will then deliver the Prevail intervention programme to employees in the intervention arm of the RCT (in groups of 10–20 employees). Employees and managers in the EAU Control group will not receive this intervention.

The Prevail-Managers programme teaches managers the skills of active problem-solving interventions, formulation-based approaches to intervention, and co-production of solution-focussed management in order to support and intervene with staff suffering from, or at risk of, developing a CMD. The philosophy behind this managerial intervention is that mental health difficulties do not occur in a social vacuum and that if staff and their managers can be taught evidence-based active problem-solving interventions and the methodology of co-production, this should greatly enhance their ability to remain in the workplace and be resilient to negative outcomes of poor mental health. Consistent with this, Gilbreath and Benson [17] found that line managers play a crucial role in employees’ quality of experience in the workplace and that the behavior of managers predicted the outcome of mental health and psychiatric disorder over and above variables such as age of employee and level of social support at home. Gilbreath and Benson [17] concluded that supervisor and managerial behavior is an important determinant of employees’ psychological well-being and should not be neglected in psychological interventions and research that attempts to improve work-place mental health. We therefore felt it important that if the Prevail intervention was successful in facilitating disclosure of mental health difficulties and help-seeking by staff experiencing CMD that it was important that the managers were facilitated to learn skills to respond effectively to this. The Prevail-Managers’ intervention programme will be delivered by our team (Gray and Snowden) to the 40 managers randomly allocated to the intervention arm of the study (in groups of approximately 20 per session).

### Employment as usual

The DVLA currently provides training courses on mental health, flexible working hours, confidential help lines, advice and a short term counselling service. This includes a 24-h Employee Assistance Programme providing confidential help and advice. DVLA also engage with organisations such as Public Health Wales and Time to Change Wales to provide a program on managing mental health issues, including a ‘brief intervention’ for alcohol problems and advice and guidance on coping with bereavement and train mental health champions to support staff experiencing mental health difficulties. Education and support is also provided by the Dementia Friends and Carers Associations, alongside the provision of an Occupational Health program.

### Procedures

Data will be collected from employment records, and by paper questionnaires handed out to individuals at the appropriate time points during the RCT evaluation (before commencement of the intervention, post Prevail-Staff and Prevail-Manager intervention programmes, and at 12 months follow-up). Data will be collected in three waves.

#### Wave 1

This will occur 1–2 weeks prior to the Prevail intervention for the Active group, with data collection for the Control group being yoked to this (but with participants and researchers being blind to which group each cohort of participants are in). Its aim is to provide baseline measures of levels of stigma and help-seeking behavior, and information about current well-being and psychological function.

#### Wave 2

This will occur approximately 4 weeks after the participant has engaged in the Prevail intervention for the Active group, with data collection for the Control group being yoked to this. Its aim is to examine if the Prevail intervention is able to change attitudes about mental health, improve help-seeking, and improve psychological well-being and levels of function.

#### Wave 3

This will occur 12 months after the Prevail intervention for the Active group, with data collection for the Control group being yoked to this. Its aim is to examine if any gains shown following engagement with the Prevail intervention in attitudinal change, mental health, and well-being are able to be sustained over a 12-month period and if the Prevail programme is able to decrease levels of absenteeism and presenteeism.

### Primary outcome measures

#### Mental health literacy and stigma

The Stigma and Self Stigma scale (SASS Docksey AE, Gray NS, Davies H, Snowden RJ: Attitudes to Mental Health Problems in the Workplace: The Development of Measures of Stigma and their Relationship to Help-seeking, Mental Health Problems, and Absenteeism, submitted) is a 42 item questionnaire that measures attitudes towards mental health problems and includes the sub-scales of stigma to others, social distance, anticipated stigma, self-stigma, avoidant coping, and lack of help-seeking. The SASS also contains items related to social desirability that is not related to mental health issues, in order to identify individuals who are giving an overly positive view of themselves [18]. Participants respond to each statement using a four-point Likert scale (strongly agree, agree, neither agree or disagree, disagree, strongly disagree).

#### Registered sick leave

Sick leave will be assessed in two ways. Our primary measure of sick leave will be records kept by the Human Resources department of the DVLA. Days of sick leave in the 12 months pre and post the Prevail intervention programme for employees in the Active intervention and EAU Control groups will be coded into “mental health reasons”, “physical health reasons” or “other” by raters blind to the group assignment of the employees.

### Secondary outcome measures

#### Self-reported sick leave

Self-reported sick leave will be assessed by asking about staff members’ estimated total number of days sick leave in the last 12 months on a seven-point scale (none, 1, 2–3, 4–10, 11–20, 21–30, 30+) and this will be repeated for sick leave separated into absences due to physical health reasons and absences due to mental health reasons.

#### Work performance/Presenteeism

Self-reported work performance will be assessed via an adaptation of the Health and Work Performance Questionnaire (HPQ [19];) using a four point Likert scale, ranging from 0 to 3 rating staff member’s work performance over the past year; comparisons of this to workers who do a similar job; and comparisons to the staff member’s usual performance. This will be followed by questions asking to what extent a physical health problem or a mental health problem affected their work performance. Staff will be asked if they have been mentally unwell whilst working and, if so, will be asked to repeat the previous questions but to respond specifically to their estimated work performance during the time that they were unwell.

#### Work and social adjustment scale

The WSAS [20] measures impairment in functioning due to a common mental disorder. It consists of five questions (e.g., “because of my [disorder], my ability to form and maintain close relationships with others, including those I live with, is impaired.”) that are answered on an eight-point scale (0 indicates no impairment at all and 8 indicates very severe impairment).

#### General anxiety disorder assessment-7

The GAD-7 [21] measures symptoms of general anxiety. Participants rate how often they have been bothered by the seven problems (e.g., “Trouble relaxing.”) over the past 2 weeks on a four-point scale (not at all, several days, more than half the days, nearly every day).

#### Patient health Questionnaire-9

The PHQ-9 [22] measures the severity of depression. The PHQ-9 is a nine item depression scale based on the diagnostic criteria for major depressive disorder. Participants rate how often they have been bothered by the nine problems listed (e.g., “Poor appetite or overeating.”) over the past 2 weeks on a four-point scale (not at all, several days, more than half the days, nearly every day).

#### Kessler psychological distress scale (K6)

The K6 [23] is a non-specific distress scale used as a screen for severe mental distress Participants self-report 6 symptoms: felt nervous, hopeless, restless and fidgety, worthless, depressed, and that everything was an effort. Each question is rated on a scale of ‘none of the time, a little of the time, some of the time, most of the time, all of the time’ (with scores of 0–4 being awarded to each respectively). Responses to the 6 items of the K6 are summed to yield a score of 0–24. Severe mental distress is defined as a K6 score of > = 13.

#### EQ-5D-5 l

The EQ***-***5D-5 L [24] is a generic measure of health status which defines health in terms of five dimensions: mobility, self-care, usual activities, pain or discomfort, and anxiety and depression. For each of the five items five descriptions are offered (e.g., I have no problems washing of dressing, to I am unable to wash or dress myself). Participants rate which description best describes their health today.

### Evaluation of ethical issues

#### Randomization

The study has a two-armed cluster randomised design that takes place at the level of the managers within the organisation. Managers will be randomly allocated into the Active intervention group or the Control group (EAU) by computer-generated random numbers. The randomisation will be stratified at the level of division within the DVLA and gender of the manager (man vs. woman) so that we achieve a similar profile of managers in each arm of the study.

#### Blinding

This study will involve a cluster-randomisation design. The employees will be randomised before they give informed consent as they will be allocated to the Control or Active intervention group depending on if their manager has been allocated to the Control or Active groups. Given the nature of the intervention it will be impossible for the managers or the employees to be blinded as to which group they are in, although the pre-evaluation assessments using psychometric tests were completed blind to group allocation (with both the researchers and the employees being blind to this). Data analysis (e.g., evaluation of number of days sick leave taken, scoring of psychometric measures) will be done by a member of the research team who will be blind as to the group allocation of the employee.

### Data analysis

Statistical analyses adapted for cluster-randomised controlled trials will be used with random effect logistic regression (for binary data) and linear regression (for continuous data) (see [25]). Should potential confounders prove to be unevenly distributed, we will adjust for these in the regression model (e.g., gender).

### Statistical power

Our cluster size is determined by the management structure of the DVLA, with each manager managing around 12 employees. A normal (non-clustered) RCT power analysis with parameters of alpha = 0.05, power of 80%, and standardised effect size of 0.30 (small effect size [26]) requires 175 per group (*N* = 350). To account for the reduction in power due to clustering we assumed an average cluster size of 12, and an intra-cluster correlation coefficient (ICC) of 0.05 and this leads to a design effect of 1.55 [27]. Hence, we require a sample size of around 271 per group (*N* = 542). Our chosen sample size (*N* = 960) exceeds this by around a factor of two as we anticipate that many employees’ data may be invalid for final data analysis due to staff leaving their current DVLA team, or not consenting to complete the psychometric measures over the three waves of evaluation (i.e. pre-Prevail, post-Prevail, and 12-months follow-up). It also allows for supplementary statistical analyses on the effectiveness of Prevail, such as a comparison between the two arms of the study for employees with a previous history of mental health problems and those without. It would by hypothesised that the effects of Prevail would be more impactful for those employees with mental health difficulties than for those employees with no such history.

### Data management

Completed questionnaires will contain no personal information to preserve anonymity. Data entry will be completed electronically. A minimum of 10% veracity checks will be completed on all data entry. All databases will be secured with password-protected access systems. All participant questionnaires will be stored in locked file cabinets in areas with limited access.

## Discussion

This study will explore the potential benefits on levels of absenteeism and presenteeism by Prevail – a workplace psychological intervention. Prevail targets mental health stigma and teaches staff the skills of low intensity psychological interventions for CMD. This includes skills of active problem-solving and how to develop a co-produced formulation plan between employees and their managers for those staff members experiencing mental health difficulties. We will explore if Prevail is successful in reducing presenteeism and sick leave due to mental health difficulties in comparison to EAU. We will also analyse the possible effects of Prevail on mental health stigma, self-stigma, anticipated stigma, help-seeking behaviour and mental health. We will investigate the experience of taking part in Prevail by the staff group to increase our understanding of what facilitates engagement with this intervention programme and the obstacles of doing so. We will conduct a health economic evaluation to explore the potential economic impact on the workplace in terms of both costs (e.g. of the Prevail programme itself, of releasing staff from their duties, of training the in-house Prevail trainers, etc) and cost benefits (e.g. reduced sick days, reduced presenteeism, a happier and more satisfied workforce) if the intervention is successful.

Sick leave and presenteeism due to CMD is a burden on individuals, their employers, and upon society. Therefore, psychological interventions that relieve this burden by reducing financial costs, improving mental health and psychological well-being, and improving productivity are in high demand. The cost/benefit of low intensity psychological interventions and active problem-solving frameworks have had mixed results in the past and a more rigorous examination of the effectiveness of these intervention programmes within the workplace is required.

## Data Availability

The datasets generated during this research and/or analysed following completion of the current study will be stored in a publically available repository such as Mendeley. The data shared will be an anonymised SPSS database that contains the item by item scores from the psychometric measures as well as the scale scores and demographic information. The data will be published at the time of submission of the paper describing the results of the RCT and will remain available indefinitely. Access will be open to anyone via the usual access to Mendeley.

## Abbreviations

CMD: Common mental disorder
EQ-5D-5 L: EuroQol- 5 Dimension – 5 levels
GAD-7: General Anxiety Disorder Assessment
HPQ: Health and Work Performance Questionnaire
PHQ-9: Patient Health Questionnaire
SASS: Stigma and Self-stigma Scale
WSAS: Work and Social Adjustment Scale
